# Group A Rotavirus Detection and Genotype Distribution before and after Introduction of a National Immunisation Programme in Ireland: 2015–2019

**DOI:** 10.3390/pathogens9060449

**Published:** 2020-06-07

**Authors:** Zoe Yandle, Suzie Coughlan, Jonathan Dean, Gráinne Tuite, Anne Conroy, Cillian F. De Gascun

**Affiliations:** UCD National Virus Reference Laboratory, University College Dublin, Dublin 4, Ireland; suzie.coughlan@ucd.ie (S.C.); jonathan.dean@ucd.ie (J.D.); grainne.tuite@ucd.ie (G.T.); anne.conroy@ucd.ie (A.C.); cillian.degascun@ucd.ie (C.F.D.G.)

**Keywords:** gastroenteritis, rotavirus, Rotarix, pediatric, diagnostics, molecular epidemiology, G3P[8], equine-like

## Abstract

Immunisation against rotavirus infection was introduced into Ireland in December 2016. We report on the viruses causing gastroenteritis before (2015–2016) and after (2017–2019) implementation of the Rotarix vaccine, as well as changes in the diversity of circulating rotavirus genotypes. Samples from patients aged ≤ 5 years (n = 11,800) were received at the National Virus Reference Laboratory, Dublin, and tested by real-time RT-PCR for rotavirus, Rotarix, norovirus, sapovirus, astrovirus, and enteric adenovirus. Rotavirus genotyping was performed either by multiplex or hemi-nested RT-PCR, and a subset was characterised by sequence analysis. Rotavirus detection decreased by 91% in children aged 0–12 months between 2015/16 and 2018/19. Rotarix was detected in 10% of those eligible for the vaccine and was not found in those aged >7 months. Rotavirus typically peaks in March–May, but following vaccination, the seasonality became less defined. In 2015–16, G1P[8] was the most common genotype circulating; however, in 2019 G2P[4] was detected more often. Following the introduction of Rotarix, a reduction in numbers of rotavirus infections occurred, coinciding with an increase in genotype diversity, along with the first recorded detection of an equine-like G3 strain in Ireland.

## 1. Introduction

Rotavirus is a leading cause of pediatric acute gastroenteritis, causing fever, vomiting, and diarrhoea. Mortality rates are highest in low income developing countries, where it causes approximately 128,000 fatal cases per year in those under five years old [1,2]. With the availability of rotavirus vaccines, the rate of global hospitalisations due to rotavirus or acute gastroenteritis, as well as deaths due to acute gastroenteritis, has decreased [3]. In Europe, pediatric rotavirus infection results in approximately 75,000−150,000 hospitalisations annually, with 2−4 times more children seeking out-patient medical care [4]. In Ireland, average crude incidence rates were 55 per 100,000 population in the 2007–2015 period [5], with hospitalisation rates of approximately 1190 per 100,000 [6], compared to the majority of EU member states, which report rates of 300–600 per 100,000 [4].

In 2009, the World Health Organisation (WHO) recommended global rotavirus vaccination [7] and, in Europe, 13 countries include it in their universal immunisation programmes, with a further five offering the vaccine for certain risk groups, specific regions, or requiring partial payment [8]. Two licensed live-attenuated vaccines are available in Europe; the pentavalent bovine-human reassortment rotavirus vaccine, RotaTeq (Merck & Co., West Point, PA, USA), and the human monovalent vaccine, Rotarix (GlaxoSmithKline, Rixensart, Belgium). In December 2016, Rotarix was introduced into the Irish national immunisation programme, with the vaccine administered in two doses at 2 and 4 months of age. Most recent figures (Q3, 2019) show the national uptake of the vaccine is 89% [9]. Rotavirus is a notifiable disease in Ireland, and laboratory confirmed cases are reported to the Health Protection Surveillance Centre. Effectiveness of both RotaTeq and Rotarix has been well documented, with the UK, Germany, and Belgium reporting an approximate 85% reduction in the presentation of severe rotavirus disease following vaccination [10,11,12].

Rotaviruses are double stranded RNA viruses containing 11 genome segments. There are 10 groups, A–J, defined by the middle VP6 capsid antigen, [13] two of which (I and J) were recently discovered in dogs and bats, respectively [14,15]. However, in humans, the majority of infections are caused by Group A rotavirus. Classification is a binary system depending on the expression of two outer proteins; the G and P-type, encoded by VP7 and VP4, respectively. Full genome analysis (where the VP7-VP4-VP6-VP1-VP2-VP3-NSP1-NSP2-NSP3-NSP4-NSP5/6 genes of rotavirus (RV) strains are described using the abbreviations Gx-P[x]-Ix-Rx-Cx-Mx-Ax-Nx-Tx-Ex-Hx), is required to monitor the evolution of the virus and detect reassortment [16].

Despite the theoretical possibility for numerous rotavirus G/P constellations, six account for 80–90% of circulating genotypes, namely G1P[8], G2P[4], G3P[8], G4P[8], G9P[8] and G12P[8]. Distribution of these commonly detected genotypes can vary by year, country, and age [17]. Despite the natural fluctuation of genotype diversity, increasing data suggest that the changes may be due to the impact of strain-specific vaccines [18]. Both in Belgium and the UK, before immunisation, G1P[8] was the most common circulating genotype; however, following vaccination, G2P[4] has been more frequently detected [19,20]. In Finland, following the introduction of RotaTeq, G9P[8] and G12P[8] have now become the main genotypes, where, previously, G1P[8] dominated [21]. However, changes in genotype distribution also occurs in countries with no immunisation [22,23], so whether the vaccine directly leads to a change in genotype diversity remains unclear [24,25]. 

Surveillance of rotavirus genotypes has been recommended by the WHO in countries with immunisation programmes to detect and monitor strain variation and ensure vaccine effectiveness is maintained [26]. The surveillance network, EuroRotaNet, has been monitoring rotavirus diversity in 12 European countries and has reported an increase in diversity since vaccination [17,27]. As Ireland is not currently part of any European or global surveillance network, we aim to fill that current gap of knowledge.

The purpose of this study is two-fold; firstly, to report on the viruses causing gastroenteritis, including rotavirus, 2 years prior and 3 years post implementation of the Rotarix immunisation programme, and, secondly, to describe the diversity of rotavirus genotypes in Ireland.

## 2. Results

### 2.1. Sample Demographics 

Ireland has a population of 4.8 million with 36% of people living in the eastern health region, which includes Dublin, the surrounding areas, and the country’s largest children’s hospitals [28]. The National Virus Reference Laboratory (NVRL), Dublin, provides a diagnostic and reference service for all health care regions, though testing is also provided in regional hospitals.

This study analyzed the results from pediatric (≤5 years) patient samples received at the NVRL between 1 January 2015 and 31 December 2019 for the investigation of viral gastroenteritis. In total, 11,800 faecal samples were included in the analysis, 5267 (45%) from females, 6511 (55%) from males, and 22 (0.2%) for which details were not provided. Samples tested were predominantly from the eastern health region 10,644/11,800 (90%), and of these 10,180/10,644 (96%) were from a children’s hospital. Other samples were from the northern 672/11,800 (6%), western 204/11,800 (2%), midlands 139/11,800 (1%), and southern health regions 141/11,800 (1%). As vaccine history was not available for each patient, cohorts are described as vaccine-eligible, using age as a proxy for vaccination status.

During 2015 to 2019, there were 312,013 births recorded in Ireland; 159,821 males (51.2%) and 152,192 females (48.8%). To establish how representative the samples tested were, the percentage of the annual birth cohort investigated for the detection of viruses causing gastroenteritis was calculated for those aged 0–12 months in each year. In 2015, 2280/65,536 (3.5%) were tested, in 2016 2065/63,841 (3.3%) were tested, in 2017 760/61,824 (1.2%) were tested, in 2018 587/61,016 (1%) were tested, and in 2019 608/59,796 (1%) were tested.

### 2.2. Detection of Viral Pathogens 

The most frequently detected viral pathogen in 2015 and 2016 was rotavirus, followed by norovirus. Norovirus has been detected in approximately 12% of samples each year, whereas enteric adenovirus (adenovirus subgenus F), sapovirus, and astrovirus were detected in 2.8–6.3% of samples from 2015–2019 (Table 1). The number of samples with no virus detected ranged from 51.4% to 65.0%, depending on the year.

There were 1753 samples tested from vaccine-eligible children in 2017–2019, and of these 43 (2.5%) had wild-type rotavirus, 179 (10.2%) had Rotarix, 257 (14.7%) had norovirus, 113 (6.4%) had adenovirus F, 97 (5.5%) had sapovirus, and 95 (5.4%) had astrovirus detected. In this group, there were 70 dual infections and 1039 (59.3%) samples had no detectable virus.

### 2.3. Detection of Wild-Type Rotavirus

The median age of those testing positive for rotavirus in the pre-vaccine era, 2015–2016 (n = 1181), was significantly lower at 1.19 years (interquartile range (IQR) 0.64–1.85), compared to the median in the entire 3 years post-vaccine, 2017–2019 (n = 373) at 1.85 years (IQR 1.12–2.84) *p* < 0.0001 (Table 2).

In the 2015/16 pre-vaccine era, a total of 485/4345 (11.2%) children aged 0–1 year had detectable wild-type rotavirus. This compares with 12/1195 (1.0%) in the post-vaccine 2018 and 2019 era, representing a 91.1% relative decrease in the number of wild-type rotavirus detected in this age range. The 1–2-year age group showed a relative reduction of 79.1% when 2015/16 was compared with 2018/19; 444/1691 (26.3%) compared to 28/505 (5.5%), respectively. This contrasts with the 5–6-year age group, which showed an increase in the detection of rotavirus from 20/324 (6.2%) to 12/96 (12.5%). 

### 2.4. Seasonal Variation of Wild-Type Rotavirus

**Figure 1:**
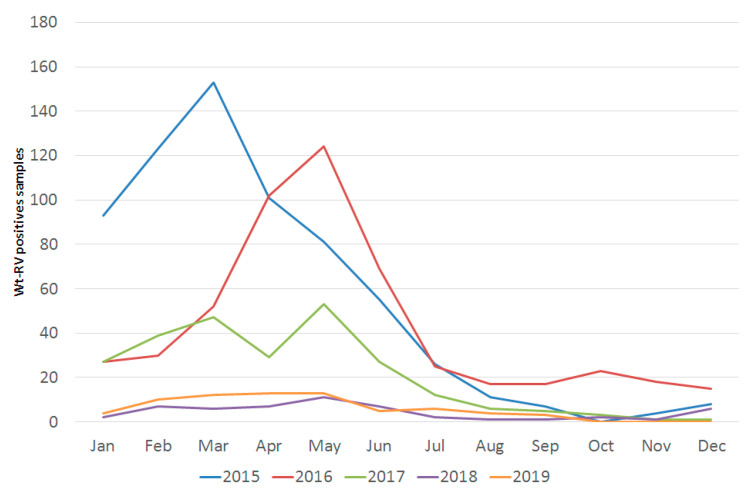
Number of wild-type rotavirus (Wt-RV) cases detected by month, 2015–2019.

Prior to vaccination, rotavirus was a seasonal infection. In 2015, the season ran from weeks 1–29, peaking in week 11; in 2016 from weeks 11–27, peaking in week 19; and in 2017 from weeks 2–30, peaking in week 11. However, in 2018 and 2019, there was no clear seasonal onset and end, and rotavirus was most frequently detected in weeks 14 and 22, respectively (Figure 1).

### 2.5. Detection of Vaccine-Derived Rotavirus (Rotarix)

Of all 3814 samples tested in 2017–2019, 1753 (46.0%) were eligible for the vaccine, and Rotarix was detected in 179/1753 (10.2%) of these (Table 1). In addition, one sample from a vaccine eligible patient was received and tested in December 2016 and found to be positive for Rotarix. In 20/180 (11.1%) of Rotarix-positive samples, another virus was detected, most commonly norovirus.

The age at which Rotarix was most frequently detected was 2 months (Table 3). Rotarix was not detected in any samples from patients older than 7 months of age.

### 2.6. Distribution of Genotypes in Ireland 

In total, 786/1554 (51%) samples with detectable wild-type rotavirus were genotyped. Of these, 728 (93%) were from the eastern health board, 33 (4%) northern, 14 (2%) western, 8 (1%) southern, and 3 (0.4%) from the midlands. No significant correlation was observed between genotype and region or age (data not shown).

As the total numbers of rotavirus cases decreased following the introduction of immunization in December 2016, the proportion of samples genotyped was increased to reliably detect significant changes. In 2015, 293/662 (44%), and in 2016, 242/519 (47%) positive samples were genotyped, while in 2017, 135/250 (54%), in 2018, 48/53 (91%), and in 2019, 68/70 (97%) were genotyped.

### 2.7. Comparison of the Genotype Diversity Pre- and Post-Vaccine

**Figure 2:**
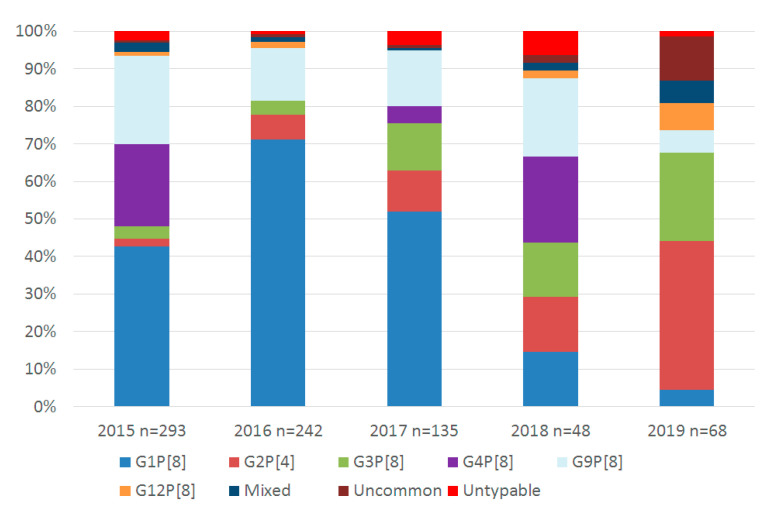
Genotype diversity in Ireland 2015–2019. Data are presented as the proportion (%) of a specific genotype compared to the total genotype results. Uncommon genotypes: <1% of total results, 2015: G9P[4] n = 2; 2016: G12P[6] n = 2; 2017: G8P[8] n = 1. 2018; G3P[4] n = 1; 2019 G2P[8] n = 1, G3P[4] n = 4, G8P[8] n = 1, G9P[4] n = 2. Mixed genotypes: those with >1 G or P-type, 2015: G1/4P[8] n = 7; 2016: G8/12P[8] n = 1, G2/3P[8] n = 1, G2/9 P[4/8] n = 1; 2017: G1/3P[8] n = 1; 2018: G1/3P[8] n = 1; 2019: G8/12P[8] n = 2, G3/12P[8] n = 1, G8P[8] n = 1, G9/12P[8] n = 1. Untypable results are those where either the G or P type was untypable.

G1P[8] was the most common genotype detected in 2015, 2016, and 2017 (Table 4). Conversely, G2P[4] was the most frequently detected genotype in 2019 (Figure 2). G3P[4], G8P[8], G9P[4], G12P[6], and G2P[8] remain uncommon genotypes in Ireland, detected in five, two, four, two, and one samples, respectively, over the 5-year period. 

### 2.8. Detection of Human and Equine-Like Rotavirus G3

**Figure 3:**
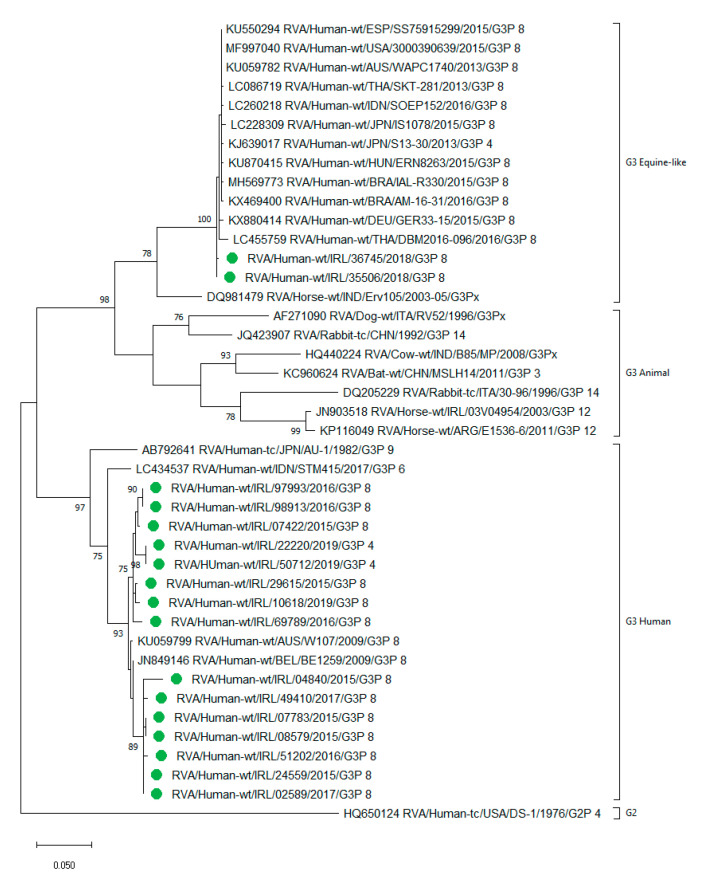
Phylogenetic tree of VP7 G3 rotavirus gene sequences. The tree was constructed by using the maximum likelihood method and the Tamura-Nei model [29]. Bootstrap values (1000 replicates) above 75% are shown. The tree is drawn to scale, with branch lengths measured in the number of substitutions per site. This analysis shows 46 nucleotide sequences; 17 from Irish strains identified in this study (colour coded with green circle) and 29 from reference strains in GenBank. Phylogenetic analyses were conducted in MEGA X [30].

G3P[8] was detected in 19/535 (4%) of genotyped samples in 2015–2016, compared to 40/251 (16%) in 2017–2019, a significant increase (p < 0.0001), whilst the uncommon G3P[4] was only detected in the post-vaccine era. Four G3 strains were detected as a mixed infection. Of the 68 G3 types (63 P[8] and 5 P[4]), 17 were selected for sequencing of the VP7 gene, which identified two G3P[8] samples from 2018, containing viruses that clustered within the equine-like G3 lineage and the remaining 15 G3 samples clustered within the human lineage (Figure 3). 

### 2.9. Genotypes Detected in Rotavirus Positive Samples from Those of Vaccine-Eligible Age

There are six common genotypes circulating in Europe, namely, G1P[8], G2P[4], G3P[8], G4P[8], G9P[8], and G12P[8] [17], and all other genotypes are considered uncommon. Of the 43 samples with detectable wild-type rotavirus and of vaccine-eligible age, 37 were genotyped by RT-PCR (Table 5). Of these, 30/37 (81.1%) had a common genotype, 4/37 (10.8%) had an uncommon genotype, 2/37 (5.4%) had a mixed infection, and one sample (2.7%) could not be fully genotyped. Ten of the 37 samples (27.0%) had G3 genotype detected (seven P[8] and three P[4]).

## 3. Discussion

This study describes the reduction in rotavirus detection following implementation of a national immunization program for all children in Ireland, as well as previously unknown data regarding the extent of genotype diversity during 2015–2019. Our study shows that the largest reduction in the detection of rotavirus occurred in those aged 0–12 months, where a relative decrease of 91% was achieved between 2015/16 and 2018/19. Although the vaccine status was unknown in detail, the effectiveness of the vaccination program has been clearly shown. Our results support the national data collated by the Health Protection Surveillance Centre (HPSC), where a crude incidence rate (CIR) of rotavirus for all age groups was 13.3 per 100,000 population in 2018, representing a decrease of 76%, compared to the mean CIR during 2008–2017 of 55.5 per 100,000 [5]. In addition, since the introduction of the vaccine, there was a reduction in visits to three large pediatric emergency departments with acute gastroenteritis, where median weekly presentations in 2017–2018 (126; interquartile range (IQR), 103–165) were lower than in 2012–2016 (160; IQR 128–214) (*p* < 0.001) [31]. Furthermore, an 86% (95% CI 79.3–90.2%) decrease in hospitalizations due to rotavirus has been reported nationally in those aged <1 year [32]. In our study, we found that the median age of wild-type rotavirus infection significantly increased in the years following vaccination, from 1.2 years in 2015 to 2.9 years in 2019 (*p* < 0.0001). This is consistent with the findings of other researchers, who also noted the later age of infection in the post-vaccine era [11,33]. In Ireland, vaccination uptake is recorded by individual General Practitioners and health care professionals which are submitted to the HPSC on a quarterly basis. Vaccine uptake data from Q1 2017 to Q3 2017 was unavailable, but the evidence suggests that this must have been suboptimal as there was little change in rotavirus detection in those aged 0–1 year in 2016 compared to 2017 (9.7% versus 7.5%, respectively). The substantial increase in rotavirus infection in the post-vaccine years in the 5–6-year age group who would not be eligible for the vaccine was also somewhat surprising. Although the number of children tested in this age group were lower in the post-vaccine compared to pre-vaccine years, the proportion of positives was almost double (6.2% versus 12.5%). The short timeframe is a limitation of this study; however, collection of data is ongoing, and it will be of interest to follow up on the impact of vaccination on rotavirus detection in all age groups in the post-vaccine era. A further finding in our data is a diminution of the characteristic rotavirus seasonal pattern, a phenomenon that has been noted by others following introduction of the rotavirus vaccine [17,34].

The live-attenuated vaccine Rotarix replicates in the gut of the recipient and is excreted, albeit at lower amounts compared to a wild-type infection [35]. We detected Rotarix in 10% of patients who were of vaccine-eligible age and, as rotavirus is notifiable in Ireland, this highlights the importance of differentiating between wild-type and vaccine-derived viruses, particularly when screening with a sensitive method, such as RT-PCR. By not excluding vaccine-derived rotavirus from diagnostic tests, there may be an over-estimation of rotavirus disease burden and unnecessary clinical intervention [36,37,38]. We identified 180 samples with detectable Rotarix, 20 (11%) of which had another virus detected, the most common being norovirus. We found norovirus to be the second most common pathogen detected after rotavirus in 2015/16, which then became the most common cause of viral gastroenteritis in our study group in the post-vaccine era. Our results are consistent with that observed in earlier studies, where norovirus is now the leading cause of viral gastroenteritis in those vaccinated for rotavirus [39,40,41]. Of note, sapovirus, astrovirus, and enteric adenovirus were detected in similar proportions over the 5-year time period and demonstrated no increase or decrease in detection rates following Rotarix introduction. Depending on the year, we report that 51–65% had no detectable viral pathogen. This apparent diagnostic gap highlights a further limitation of this study, in that it is quite possible that parallel samples were sent for the investigation of bacterial or parasitic pathogens, which are common causes of gastroenteritis [42,43]. Unfortunately, we did not have access to these results. In addition, other viruses, such as bocavirus, enterovirus, and parechoviruses, which may cause gastroenteritis, would not have been detected by our routine screening test. 

Prior to the introduction of Rotarix, we found the circulating genotypes in Ireland were comparable to other European countries, with G1P[8] being the most commonly detected. The findings of the current study are consistent with those observed in several earlier reports from samples tested in Ireland from 1995 to 2009, where it was reported that the most commonly detected genotype was G1P[8], with fluctuating levels of G2P[4], G3P[8], G4P[8], and G9P[8] [44,45,46,47,48,49]. The current study matches those findings. However, we can report that the diversity of genotypes increased in the years following the introduction of a vaccine and that, in 2018/19, G1P[8] was no longer the most common genotype. Furthermore, genotypes detected in children eligible for the vaccine was more varied than those detected in the vaccine-ineligible cohort. With regards to wild-type and vaccine rotavirus strains, they can be described in terms of being homotypic, partly heterotypic, and fully heterotypic based on the G and P proteins. For instance, the monovalent G1P[8] Rotarix vaccine is homotypic to other circulating G1P[8] strains (both proteins are the same), G12P[8] is partly heterotypic (one protein different), and G2P[4] is fully heterotypic (both G and P proteins are different) [50]. Rotarix provides exposure to G1P[8] rotavirus among infants, with protection that is likely to be higher against homotypic strains than heterotypic strains, such as G2P[4]. This suggests that natural infection leading to disease is more likely to be caused by such heterotypic strains [19,51] and that a vaccinated population could possibly drive selective pressure, increasing the likelihood of these genotypes to circulate in the community [52,53]. That being said, the monovalent vaccine Rotarix provides significant protection from G1, G2, G3, G4, and G9, and efficacy against severe G2 rotavirus gastroenteritis was as high as for other rotavirus types [54]. Clearly, the immune response to rotavirus infection is a complex issue, with a previous report suggesting that type-specific neutralizing antibodies induced by the vaccine against VP7/VP4 epitopes are not solely responsible for a protective effect [55]. The report proposes that, as there are a limited number of diverse circulating strains worldwide, these antibodies are not driving long-term selective pressure, which itself would favor antigenic drift or the emergence of novel genotypes. 

Interestingly, the genotype G12P[8] was not circulating widely at the time of Rotarix and RotaTeq vaccine development; however, it has now become established as an increasingly common genotype [17]. A large study in the USA found G12P[8] more frequently than any other rotavirus genotype in fully vaccinated children [56]. Another example of an uncommon genotype becoming more prevalent is the recently emerged equine-like G3 strain, first identified in Japan in 2013 [57] but now detected world-wide [58,59,60]. We identified, for the first time in Ireland, two samples from 2018 that clustered within the equine-like G3 lineage. A further 15 G3 samples were sequenced and all clustered in the human lineage, suggesting that the equine-like lineage has not yet become established in Ireland compared to other countries [59,61]. Of note, five of the uncommon human G3P[4] strains were also identified in our study in 2018–2019, and this strain has been detected before in Ireland in 2006/07 [48]. The detection of uncommon genotypes, along with the additional potential for zoonotic reassortment [62,63], reinforces the WHO recommendation for surveillance, emphasising the need for continued monitoring of rotavirus vaccine efficacy against emerging rotavirus.

Several important limitations need to be considered for our study group. Firstly, the results are somewhat biased due to the observational nature of the study and samples tested would have been from those with moderate to severe gastroenteritis that warranted clinical investigation. In addition, there was no denominator for the population not suffering from symptoms of viral gastroenteritis, so we are unable to calculate incidence and prevalence of rotavirus infection. Furthermore, with no access to vaccination data, it was not possible to determine vaccine effectiveness rates or describe definitive vaccine failures. However, due to the large data set (n = 11,800), we can show relative reductions in the detection of rotavirus and the changes in the diversity of circulating genotypes. The geographical distribution of samples is nationwide, although the data are skewed to some extent due to the density of the population in Dublin and the location of the main children’s hospitals, and therefore samples were predominantly from the eastern health region. It should also be noted that the number of samples tested has decreased year on year. The reason for this may be the decrease in symptomatic children or the increase in localized testing and possible availability of point of care assays. Finally, it was not possible to categorize samples as the community or hospital acquired and we could not identify samples belonging to outbreaks. 

We have shown that rotavirus continues to circulate in the pediatric population, albeit in low numbers, and this is expected to decrease further with the increasing cohort of vaccinated children. Binary genotypic classification is useful to establish circulating genotypes and can be used for reassortment studies of the VP7 and VP4 encoding genes; however, whole genome genotyping is required for a more detailed analysis of the virus. Indeed, a future aim from this ongoing study is to perform whole genome sequencing from samples in this dataset to allow identification of possible reassortment of non VP7/VP4 genes or mutation events. In addition, all samples identified as G3 will be categorized as either equine-like or of human lineage. By collaborating with clinicians at the children’s hospitals, it is hoped that any sample from a child with rotavirus with a full or partial vaccine history will be referred to the NVRL for whole genome sequencing to establish definitive strains circulating in this group of children.

In conclusion, we describe the detection and characterization of rotavirus in pediatric samples circulating in Ireland over a 5-year time period. We show that, following the introduction of Rotarix, there is a relative reduction in the number of rotavirus infections diagnosed, coinciding with an increase in genotype diversity, along with the first recorded detection of an equine-like G3 strain in Ireland.

## 4. Materials and Methods

### 4.1. Study Design

This opportunistic study presents the results of faecal samples from pediatric samples (≤5 years) investigated for viral gastroenteritis at the National Virus Reference Laboratory (NVRL), Dublin, Ireland. Test results for wild-type rotavirus, vaccine-derived rotavirus (Rotarix), norovirus, sapovirus, astrovirus, and enteric adenovirus subgenus F were obtained with genotype and sequence results, if available. Samples dated 1 January 2015 to 31 December 2019 were included in the study. Samples dated 1 January 2015 to 31 December 2016 were designated as “pre-vaccine”. The first doses of Rotarix were given for those aged 2 months from the 1 December 2016. Only one sample was received from a 2-month old in December 2016, and this patient had detectable Rotarix. Samples dated 1 January 2017 to 31 December 2019 were designated as “post-vaccine”. Routine testing for Rotarix was introduced into the NVRL from 11 December 2017. Rotavirus-positive samples received from 1 December 2016 to 10 December 2017 were tested for Rotarix, retrospectively.

### 4.2. Annual Birth Cohort in Ireland

The Central Office of Statistics provides the annual number of births in Ireland [64]. The number of annual births by year are: 2015: 65,536 (33,480 males, 32,056 females); 2016: 63,841 (32,709 males, 31,132 females); 2017: 61,824 (31,779 males, 30,045 females); 2018: 61,016 (31,298 males, 29,718 females); 2019: 59,796 (30,555 males, 29,241 females,). Data for 2018 and 2019 are provisional. The overall male: female ratio for 2015–2019 was 1.1: 1 (51.2% versus 48.8%).

### 4.3. Data Analysis

Data were extracted from the NVRL Laboratory Information Management System and analyzed in Excel. All samples were assumed to be from symptomatic patients. Samples with no date of birth recorded or duplicate samples were excluded from the study. Patients were de-identified, and the variables recorded in the database were patients’ age at sample collection, sex, sample date, geographical region, and test result(s). Geographical regions were categorized as eastern (which includes Dublin), western, southern (south and south-east), northern (north-west and north-east), and midlands (midlands and mid-west), as defined by the Health Service Executive areas used by the Health Protection Surveillance Centre [5].

### 4.4. Sampling Strategy for Genotyping and Sequencing of Samples

To determine the sample size required to reliably detect a change in genotype frequency, a sample size calculator was used (CL95%, www.openepi.com), and then a random selection of wild-type rotavirus samples was selected for genotyping. In addition, all uncommon genotypes and a random subset of genotyped samples was selected for sequencing of VP7 and VP4 genes. A subset of those identified as G3 and were sequenced and analyzed to determine a human or equine-like lineage. 

### 4.5. Seasonality

Seasonal onset, peak, and end were calculated: Onset: First of 2 consecutive weeks, where the median percentage of positive results was >10%. Peak: Week with the highest proportion of positive samples. End: Last of two consecutive weeks, where the median percentage was <10%. Denominator: total samples tested; numerator: the number of positive samples.

### 4.6. Vaccine Eligibility

The vaccine status was unknown, and patients were categorised by vaccine eligibility. Vaccine-eligible samples were those born after 1 October 2016 and were ≥2 months of age. Vaccine-ineligible samples were those born after 1 October 2016 and were <2 months of age or were born prior to 1 October 2016 and were aged 0–5 years of age. 

### 4.7. Laboratory Methods

Upon receipt into the laboratory, approximately 20% *w*/*v* suspension of the fecal sample was prepared in 400 µL Stool Transport and Recovery Buffer (Roche) and 400 µL external lysis buffer (Roche). A total of 450 µL of the suspension was extracted by Roche MagNAPure 96 and eluted into 100 µL. During extraction, Brome Mosaic Virus RNA (University of Indiana) was added as an internal control (IC) at 1 pg/µL to the sample prior to extraction. The eluates were tested in five one-step RT-PCR assays, as previously described [65,66,67,68,69,70]. Briefly, eluates were tested in a 25 μL or 10 µL reaction mixture (depending on the 96- or 384-well format, respectively), containing 2× Superscript™ III Platinum One-Step qRT-PCR mix (Invitrogen), as per product insert. Final concentrations of primers and probes ranged from 80 nM to 400 nM, depending on the target. Each sample eluate was tested by five (RT-)PCR reactions, namely: (i) norovirus G1/G2/IC; (ii) adenovirus F/pan-rotavirus/IC; (iii) Rotarix; iv) astrovirus/IC; v) sapovirus/IC. Amplification was performed on the ABI 7500 Fast (96-well format) or the ABI Viia7 (384-well format) instrument under the following conditions: 15 mins 50 °C, 2 mins 95 °C, 38 cycles of 15 secs 95 and 30 secs 60 °C (56 °C for norovirus). Amplification data was collected and analyzed with Sequence Detection Software version 2.3 or the Viia7 software version 1.2.1 (both from Applied Biosystems).

Genotyping was either by a multiplex RT-PCR [71,72] or by hemi-nested RT-PCR, as described previously [73], with fragment visualization and size determination performed on the TapeStation (Agilent software, version 2200). Samples with an indeterminate G or P type were tested by both methods before being categorized as untypable. A selection of genotypes were confirmed by Sanger sequencing of the VP7 and VP4 genes, using previously described methods [73] on the ABI 3500Dx genetic analyzer (Applied Biosystems) and typed using the RotaC typing tool [74] or by the Basic Local Alignment and Search Tool, BLAST (http://www.ncbi.nlm.nih.gov/blast/Blast.cgi). G3 VP7 sequences (450 nucleotides) were aligned with appropriate reference sequences using ClustalW. Phylogenetic analyses were conducted in MEGA X [30] using the maximum likelihood method, with 1000 bootstrap replicates, based on the Tamura-Nei model [29]. This model was selected as it generated the lowest Bayesian information criterion (BIC) score in MEGA X.

### 4.8. GenBank Accession Numbers 

Partial VP7 fragments of the equine-like G3 strains identified in this study were deposited in GenBank under the following accession numbers: strains; MT475885 and MT4758866, whereas the human-lineage G3 VP7 fragments were MT537569-537583.

### 4.9. Statistical Analysis

The study was observational and therefore most data presented was descriptive. The median age of rotavirus infection in the pre- and post-vaccination groups was compared using Mann–Whitney *U* test. The Chi-square test for proportions was used to compare genotypes in the pre- and post-vaccination groups. *P* values for both tests of ≤0.05 were considered statistically significant. Confidence intervals (95%) were calculated using the Wilson method for a proportion of the genotypes detected. Statistical analysis was performed using SPSS 26 (IBM Corp; Armonk, NY, USA) software or www.openepi.com.

### 4.10. Ethical Statement

All procedures performed in studies involving human participants were in accordance with the ethical standards of the institutional and/or research committee and with the 1964 Helsinki declaration and its later amendments or comparable standards. This study was approved for ethical exemption by University College, Dublin LS-E-17-09.

## Figures and Tables

**Table 1:** Laboratory results for the investigation of viral gastroenteritis in 11,800 samples tested at the National Virus Reference Laboratory (NVRL), aged 0–5 years in 2015–2019.

	Results (%) by Year					Total
Virus Detected	2015	2016	2017	2018	2019	
Rotavirus-wild-type	662 (15.03)	519 (13.09)	250 (15.49)	53 (4.42)	70 (5.93)	1554
Rotavirus-Rotarix	0 (0.00)	1 (0.03)	61 (3.78)	49 (4.08)	69 (5.84)	180
Norovirus	482 (10.94)	492 (12.41)	210 (13.01)	158 (13.17)	141 (11.94)	1483
Adenovirus F	156 (3.54)	155 (3.91)	101 (6.26)	64 (5.33)	47 (3.98)	523
Sapovirus	202 (4.59)	167 (4.21)	85 (5.27)	72 (6.00)	33 (2.79)	559
Astrovirus	197 (4.47)	121 (3.05)	77 (4.77)	68 (5.67)	53 (4.49)	516
No virus detected	2705 (61.42)	2511 (63.31)	830 (51.43)	736 (61.33)	768 (65.03)	7550
Total samples tested	4199	3787	1499	1159	1156	
Total results	4404 ^a^	3966 ^b^	1614 ^c^	1200 ^d^	1181 ^e^	

^a^ 185 dual infections, 10 triple infections ^b^ 154 dual infections, 11 triple infections, 1 quadruple infection, ^c^ 93 dual infections, 8 triple infections, 2 quadruple infections ^d^ 41 dual infections ^e^ 25 dual infections. Additional viruses detected in Rotarix samples, 2017: norovirus n = 3, adenovirus F n = 1, astrovirus n = 2; 2018: norovirus n = 8, adenovirus F n = 1; 2019: norovirus n = 3, sapovirus n = 1, astrovirus n = 1.

**Table 2:** Number of wild-type rotavirus positive cases by age group in the pre-vaccine years (2015–2016) compared to the post-vaccine years (2017–2019).

	Year	Number of Wild-Type Rotavirus Positive Samples/Total Number of Samples Tested (%)							Median Age (IQR)
		0–1 Year	1–2 Years	2–3 Years	3–4 Years	4–5 Years	5–6 Years	Total	
Pre-vaccine	2015	285/2280 (12.5)	227/899 (25.3)	90/413 (21.8)	35/213 (16.4)	17/213 (8.0)	8/181 (4.4)	662/4199 (15.8)	1.17 (0.5-1.9)
	2016	200/2065 (9.7)	217/792 (27.4)	55/427 (12.9)	24/224 (10.7)	11/136 (8.1)	12/143 (8.4)	519/3787 (13.7)	1.22 (0.8-1.8)
Post-vaccine	2017	57/760 (7.5)	110/374 (29.4)	49/144 (34.0)	19/105 (18.1)	6/55 (10.9)	9/61 (14.8)	250/1499 (16.7)	1.59 (1.0–2.4)
	2018	8/587 (1.4)	12/272 (4.4)	23/125 (18.4)	2/62 (3.2)	3/59 (5.1)	5/54 (9.2)	53/1159 (4.6)	2.24 (1.6–2.9)
	2019	4/608 (0.7)	16/233 (6.9)	16/129 (12.4)	21/86 (24.4)	6/58 (10.3)	7/42 (16.7)	70/1156 (6.1)	2.90 (1.9–3.5)

Interquartile range (IQR).

**Table 3:** Detection of Rotarix by age.

	Age					
	2 mts	3 mts	4 mts	5 mts	6 mts	7 mts
Rotarix detected/total number Rotarix detected (%)	99/180 (55.0)	41/180 (22.8)	26/180 (14.4)	11/180 (6.1)	2/180 (1.1)	1/180 (0.6)

**Table 4:** Comparison of genotype diversity between the pre-vaccine (2015–2016) and post-vaccine (2017–2019) eras. Confidence interval (CI) significance 0.95. Comparison of the genotype proportion between pre- and post-vaccine year groups by Chi-square *p* < 0.05.

	Pre-Vaccinen (%)		Post-Vaccinen (%)			Pre-Vaccine Data Combined		Post-Vaccine Data Combined		Pre vs. Post
Genotype	2015	2016	2017	2018	2019	n (%)	CI 95%	n (%)	CI 95%	*p* =
G1P[8]	125 (42.7)	172 (71.1)	70 (51.9)	7 (14.6)	3 (4.4)	297 (55.5)	51.3–59.7	80 (31.9)	26.4–37.9	<0.0001
G2P[4]	6 (2.0)	16 (6.6)	15 (11.1)	7 (14.6)	27 (39.7)	22 (4.1)	2.7–6.2	49 (19.5)	15.1–24.9	<0.0001
G3P[8]	10 (3.4)	9 (3.7)	17 (12.6)	7 (14.6)	16 (23.5)	19 (3.6)	2.3–5.5	40 (15.9)	11.9–21.0	<0.0001
G4P[8]	64 (21.8)	0 (0.0)	6 (4.4)	11 (23.0)	0 (0.0)	64 (12.0)	9.5–15.0	17 (6.8)	4.3–10.6	0.0257
G9P[8]	69 (23.5)	34 (14.1)	20 (14.8)	10 (20.9)	4 (5.9)	103 (19.3)	16.1–22.8	34 (13.6)	9.9–18.3	0.0493
G12P[8]	3 (1.0)	4 (1.7)	0 (0.0)	1 (2.1)	5 (7.4)	7 (1.3)	0.1–2.7	6 (2.4)	1.1–5.1	0.2675
Mixed	7 (2.4)	3 (1.2)	1 (0.7)	1 (2.1)	4 (5.9)	10 (1.9)	1–3.4	6 (2.4)	1.1–5.1	0.0014
Uncommon	2 (0.7)	2 (0.8)	1 (0.7)	1 (2.1)	8 (11.8)	4 (0.8)	0.03–1.9	10 (4.0)	2.2–7.2	0.6295
Untypable	7 (2.4)	2 (0.8)	5 (3.7)	3 (6.3)	1 (1.5)	9 (1.7)	0.9–3.2	9 (3.6)	1.9–6.7	n/a
Total	293 (100)	242 (100)	135 (100)	48 (100)	68 (100)	535 (100)	n/a	251 (100)	n/a	n/a

**Table 5:** Wild-type rotavirus genotypes detected in the age group eligible for the vaccine. Six additional samples had detectable wild-type rotavirus but were unavailable for genotyping.

Genotype	Classification of Genotype	Number of Samples (%)
G1P[8]	Common	5 (13.5)
G2P[4]	Common	11 (29.7)
G3P[8]	Common	6 (16.2)
G4P[8]	Common	1 (2.7)
G9P[8]	Common	4 (10.8)
G12P[8]	Common	3 (8.1)
G3P[4]	Uncommon	3 (8.1)
G9P[4]	Uncommon	1 (2.7)
G1/3 P[8]	Mixed	1 (2.7)
G9/12 P[8]	Mixed	1 (2.7)
G9 P untypable	Untypable	1 (2.7)
Total		37 (100)
